# A Sustainable and Eco-Friendly Membrane for PEM Fuel Cells Using Bacterial Cellulose

**DOI:** 10.3390/polym16213017

**Published:** 2024-10-28

**Authors:** Xiaozhen Yang, Lin Huang, Qiang Deng, Weifu Dong

**Affiliations:** The Key Laboratory of Synthetic and Biological Colloids, Ministry of Education, School of Chemical and Material Engineering, Jiangnan University, 1800 Lihu Road, Wuxi 214122, China; yangxz@stu.jiangnan.edu.cn (X.Y.); huanglin@jiangnan.edu.cn (L.H.); dengqiang@stu.jiangnan.edu.cn (Q.D.)

**Keywords:** bacterial cellulose, alkaline proton carrier, PEM, fuel cells, proton conductivity, fully bio-based membrane

## Abstract

Bacterial cellulose (BC) is an advantageous polymer due to its renewable nature, low cost, environmental compatibility, biocompatibility, biodegradability, chemical stability, and ease of modification. With these advantages, BC is an interesting candidate for the development of novel eco-friendly materials for proton-exchange membrane (PEM) applications. However, its practical applications have been limited by its relatively high dispersion in water, which usually occurs during the operation of proton-exchange membrane fuel cells (PEMFCs). In addition, the proton conductivity of bacterial cellulose is poor. In this study, functionalized BC modified with 3-aminopropyltriethoxysilane (APTES) was prepared using a solvent casting method to enhance its performance. The results showed that the water stability of the modified BC membrane was significantly improved, with the contact angle increasing from 54.9° to 103.3°. Furthermore, the optimum ratio of BC and APTES was used to prepare a proton-exchange membrane with a maximum proton conductivity of 62.2 mS/cm, which exhibited a power generation performance of 4.85 mW/cm^2^ in PEMFCs. It is worth mentioning that modified BC membranes obtained by combining an alkaline proton carrier (-NH_2_) with BC have rarely been reported. As fully bio-based conductive membranes for PEMFCs, they have the potential to be a low-cost, eco-friendly, and degradable alternative to expensive, ecologically problematic fluoric ionomers in short-term or disposable applications, such as biodegradable electronics and portable power supplies.

## 1. Introduction

Proton-exchange membrane fuel cells (PEMFCs) are an important pathway for hydrogen energy applications in the context of carbon neutrality [1]. As a green and environmentally friendly energy source, proton-exchange membrane fuel cells are electrochemical reaction devices that produce water through the reaction of hydrogen and oxygen [2]. In a PEMFC, hydrogen is oxidized at the anode and oxygen is reduced at the cathode in the presence of a noble metal (e.g., platinum) electrocatalyst [3,4]. In this context, proton-exchange membranes (PEMs) serve as solid polymer electrolytes, facilitating ion exchange between the two electrodes. It is necessary for PEMs to have good proton conductivity, electronic insulation, mechanical properties, and gas barrier properties to separate the anode and cathode sides.

Sulfonated fluoropolymers have dominated the market since their discovery in the 1960s [5]. Currently, the commercial Nafion membrane, with a proton conductivity of 100 mS/cm in a hydrated state, is commonly used in PEMs. The high proton conductivity of the Nafion membrane is due to phase separation between the hydrophobic and hydrophilic domains, resulting in a permeable ion-conduction channel that provides effective proton transport [6]. However, the Nafion membrane’s raw materials contain fluorine; additionally, its manufacturing process is complex, it is difficult to recycle, and its price is extremely high. Therefore, it is of great significance to study sustainable, low-cost, and fluorine-free proton-conducting polymers for PEMFCs.

In addition, the effective utilization of biomass resources has always been a crucial problem for the sustainable development of society, which is also an indispensable driving force for China’s dual carbon goals and similar goals globally. As the main component of plants and trees, cellulose is the most abundant material resource in the world [7]. As a subclass of cellulose, nanocellulose is widely used in the energy field due to its inherent characteristics (e.g., rich hydroxyl groups, easy chemical modification, high mechanical strength, and excellent barrier properties) [8].

Many efforts have been made to develop alternative PEMs for PEMFCs. Non-fluorinated hydrocarbon-based aromatic polymers are materials of considerable interest due to their low cost, flexibility in terms of synthesis and molecular design, film formation tendency, and thermomechanical properties, which enable their use in a variety of applications, including rechargeable batteries, fuel cells, and separation science [9]. Aromatic polymers, such as sulfonated forms of polyimides, poly(ether ether ketone) (PEEK), poly(arylene ether sulfone), poly(ether ketone), and polybenzimidazole are being widely studied as base skeletons for PEM fabrication [10,11,12]. However, hydrocarbon-based aromatic polymers are still inevitably used in whole or in part.

The applications of nanocellulose in PEMs have mainly involved its use as a reinforcing agent mixed with sulfonated polymers (e.g., Nafion and SPEEK) [2,13,14,15,16]. However, the use of fluorides or hydrocarbons has not yet been completely avoided. Therefore, the development of fully bio-based conductive membranes has advantages over fluorinated and hydrocarbon PEMs, particularly for disposable or short-term applications. Many studies in the literature have focused on the acidification of nanocellulose, including sulfonic acid (-SO_3_H)-modified cellulose nanocrystals [17,18,19], carboxylic acid (-COOH) chemically modified cellulose nanofibers [20], and the treatment of bacterial cellulose (BC) with sulfonic acid or phosphoric acid [21,22].

BC is a kind of cellulose material with a 3D nanostructure morphology formed of nanofibers with diameters of 20–100 nm. Surface -OH groups are contained in BC, which has the characteristics of high crystallinity, high tensile strength, strong hydrophilicity, and low gas permeability. Water is always produced during the operation of PEMFCs, so PEMs must be stable in water. BC is highly dispersed in water and has limited proton conductivity, so it needs to be modified for use in PEMFCs. Although conventional acidic proton carriers (e.g., -SO_3_H and -COOH) can dissociate to form a large number of protons, resulting in high proton conductivity, the highly dissociated groups make the structure of the membrane vulnerable to damage. Additionally, the significant swelling of the membrane results in decreased structural stability [23]. In addition, under conditions of high temperature and water shortage, the combination of acidic proton carriers and BC is likely to lead to cellulose carbonization, which leads to membrane breakdown [24]. However, alkaline proton carriers (such as -NH_2_) may alleviate this problem. To date, no research reports have been published on the application of amino-modified BC in PEMFCs [17,19,20,22,25,26,27] (as shown in Table S2 in the Supporting Information).

In this study, we utilized 3-aminopropyltriethylsilane (APTES), which is inexpensive and readily available, to modify BC to solve the problem of its high dispersion in water. The experimental results demonstrated that the modified BC exhibited good proton conductivity and stability in water due to the presence of APTES with alkaline proton carriers (-NH_2_). Ultimately, the application of the prepared BC membrane in fuel cells was validated by constructing a membrane electrode assembly and testing the polarization curves of a single cell.

## 2. Materials and Methods

### 2.1. Materials

The BC dispersion used in this study was purchased from Qihong Technology Co., Ltd (12 Jiangan Road, Qixing District, Guilin, Guangxi, China). Fibrous nanomaterial with an ultra-high aspect ratio was obtained via the fermentation of gluconobacter acetate with sugar as the raw material. Its appearance was that of a milky white gel, its cellulose dry weight content was 0.8 wt%, the fiber diameter was 50–100 nm, the fiber length was 10–20 μm, and the surface functional groups were -OH. APTES (C_9_H_23_NO_3_Si, 98%), glacial acetic acid (C_2_H_4_O_2_), and anhydrous ethanol (C_2_H_6_O) were purchased from Macklin Reagent Co., Ltd (Shanghai, China). All reagents were used directly without further purification.

### 2.2. Preparation of Unmodified-BC and Modified-BC Membranes

**Figure 1:**
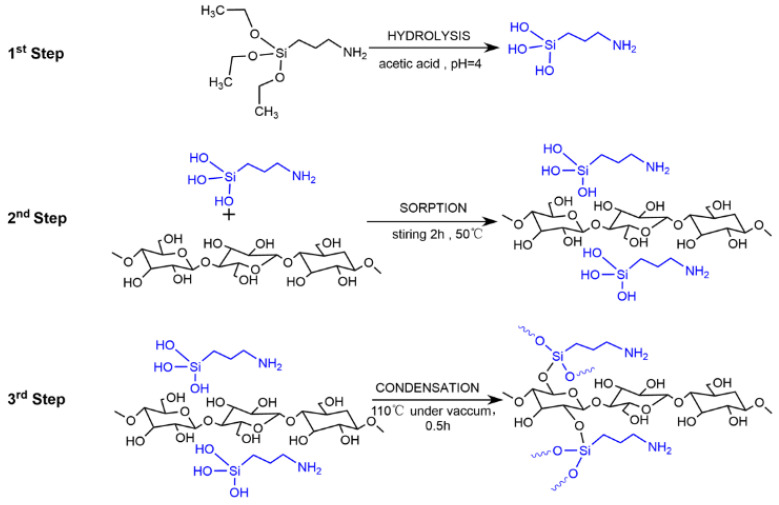
Schematic diagram of BC modification with APTES.

A schematic diagram of BC modification with APTES is shown in Figure 1. APTES was pre-hydrolyzed at room temperature. In brief, 5 wt% of each silane was dissolved in ethanol solution. The pH of the APTES was adjusted to ~4.0 by adding diluted acetic acid (in ethanol), and the mixture was continuously stirred for 4 h. When the pH was stable, the 3-aminopropyltriethylsilane solution (prepared as above) was immediately added to the BC dispersion at different mass ratios (pure silane/dried bacterial cellulose ratios of 0.1–1:1.0 by wt%). The mixture was magnetically stirred at 50 °C for 2 h. The casting solution was poured into an ~85 mm diameter plastic surface dish and dried at 40 °C for 24 h. After the solvent was removed, the BC membrane was annealed in a vacuum-drying oven at 110 °C for 0.5 h, then washed three times with 95% ethanol aqueous solution and dried at 40 °C for 12 h. An original BC membrane was prepared by using the same manufacturing method, except for the addition of APTES; this was referred to as the Unmodified-BC membrane. Membrane samples were obtained and characterized by means of Fourier transform infrared spectroscopy (Figure S1 in the Supporting Information). The new infrared peak at 1550 cm^−1^ for the modified membrane samples, as compared to the Unmodified-BC membrane, can be attributed to N-H bending vibration in APTES. A suitable degree of silanization is beneficial to the proton conductivity of the membranes. The optimal conductivity of the membranes was selected for further research. The final membrane obtained at the most suitable ratio (pure silane/dried bacterial cellulose ratio of 0.825:1.0 by wt%) was referred to as the Modified-BC membrane. The thickness of the Unmodified-BC and Modified-BC membranes was 35 ± 5 μm in dry form after their preparation.

### 2.3. Contact Angle Testing

The static water contact angles of the membranes before and after chemical modification were measured using a static contact angle measuring instrument (OCA40, DataPhysics Instruments GmbH, Filderstadt, Germany). The droplet size was controlled using a syringe. At room temperature, 2 μL of water droplets were deposited on the membrane with a micropipette, and the contact angle of each membrane was measured 10 s after water droplet deposition to ensure that the water droplets reached equilibrium. To ensure the reliability of the data, each membrane was tested in three equal parts, and the final contact angle was calculated as the average value.

### 2.4. Water Uptake and Swelling Ratio Testing

The water uptake and swelling ratio of the membranes were determined by measuring the variations in weight and length before and after hydration. The mass and length were measured using an analytical balance (±0.1 mg) and a micrometer (±1 μm), respectively. All samples (1 cm × 3 cm) were first vacuum-dried at 80 °C for 8 h and then weighed immediately after cooling. Subsequently, the samples were submerged in water at different temperatures for 1 h. Finally, the samples were taken out, and any excess surface water was removed using air-laid paper. The water uptake was calculated according to the following formula: $water\;uptake\left(\%\right)=\left(m_{wet}-m_{dry}\right)/m_{dry}\times 100\%$, where $m_{dry}$ is the mass of the dry membrane and $m_{wet}$ is the mass of the wet membrane after water saturation. The swelling ratio was calculated according to the following formula:$\;Swelling\;ratio(\%)=\left(V_{wet}-V_{dry}\right)/V_{dry}\times 100\%$, where $V_{dry}$ is the volume of the dry membrane and $L_{wet}\;$is the volume of the wet membrane after water saturation.

### 2.5. SEM and EDS Analysis

The morphology of the membranes was investigated by means of field-emission scanning electron microscopy (S-4800, Hitachi Limited, Tokyo, Japan). The surface and cross-sectional morphology changes in the membranes before and after chemical modification were analyzed. All the samples were sputter-coated with Au to eliminate the charging effect, reduce the vacuum of the sample chamber, and introduce molecules with a positive charge.

### 2.6. Raman Spectroscopy Analysis

The membranes were characterized by means of Fourier transform infrared spectroscopy (Nicolet iS50 FT-IR, Thermo Fisher Scientific, Waltham, MA, USA) in attenuated total reflection (ATR) mode. A total of 32 scans were performed on each sample, with a scan range of 4000 to 500 cm^−1^ and a scan resolution of 4 cm^−1^.

### 2.7. XRD Analysis

The samples were tested using an X-ray diffractometer (Bruker D8, Karlsruhe, Germany) equipped with a Cu target to study the effect of chemical modification on the crystallinity of the Unmodified-BC and Modified-BC membranes. The 2θ angle scanning range was 10~40°, and the step size was 0.02°.

### 2.8. XPS Analysis

The chemical composition and bonding environment of the Modified-BC membrane were evaluated by means of X-ray photoelectron spectroscopy measurements (Kratos Axis supra, Manchester, UK) using monochromatized Al Ka radiation (hv = 1486.6 eV, 150 W) as the X-ray source with a base pressure of 10^−9^ torr. A pass energy of 160 eV and a step size of 1 eV were used to acquire the survey scan spectra. A pass energy of 40 eV and a step size of 0.1 eV were utilized to acquire narrow-region scans. The hybrid lens mode was used in both cases. The analyzed area of all XPS spectra was 300 × 700 ${\mu m}^{2}$. To isolate the sample from the sample rod, a charge neutralizer was used during sample installation. All spectra were calibrated using C 1 s (284.8 eV).

### 2.9. SAXS Analysis

The samples were tested by means of small-angle X-ray scattering (Xeuss 3.0, Grenoble, France) equipped with a Cu target to study the nano–microphase separation proton channel of the Modified-BC membrane. The sample-to-detector distance was 1500 mm.

### 2.10. Proton Conductivity Testing

The proton conductivity of the membranes was studied using an electro-chemistry workstation (PARSTAT2273, AMETEK, Prinston, NJ, USA) with electrochemical impedance spectroscopy. The scanning frequency range was 1 MHz~10 Hz, and the disturbance voltage was 100 mV. Firstly, each membrane was cut to a size of 1 cm × 3 cm, fixed in a test fixture (Figure S2 in the Supporting Information), and placed in an environment of 45~95 °C at 100% relative humidity for 1 h to equilibrate. Then, the impedance spectrum was measured using the PARSTAT2273 workstation. Finally, the data were fitted to an equivalent circuit model (1) [28], and the resistance (R) was determined from the high-frequency intercept. The proton conductivity was calculated according to Equation (2).

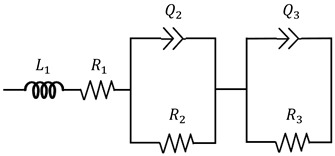
(1)

Here, $L_{1}$ is the inductance; $R_{1}$, $R_{2}$, and $R_{3}$ are the resistance values; and $Q_{2}$ and $Q_{3}$ are the constant-phase elements.

(2) σ=(L/R)×A

Here, L is the distance between two electrodes, unit: cm; R is the ohmic resistance of the measured membrane sample, unit: Ω; and A is the cross-sectional area of the membrane perpendicular to the electrode plate, unit: cm^2^.

### 2.11. Fuel Cell Testing

The polarization curve of a membrane electrode assembly (MEA) was measured at 75~95 °C at 100% relative humidity using a fuel cell test system (HOST-120A, Hydrogen Core Technology (Wuxi) Co., Ltd., Wuxi, China). The anode and cathode of the MEA were supplied with hydrogen gas and air at flow rates of 100–200 mL/min and 300–600 mL/min, respectively. Both the anode and cathode back pressures were set to 0.1 MPa.

## 3. Results and Discussion

### 3.1. Water Stability

A PEM typically operates under wet conditions and generates water at the cathode, so its stability in water is essential. As shown in Figure S3 in the Supporting Information, the original BC membrane was intact in the dry state. After infiltration in deionized water for only 5 min, membrane rupture was observed, apparently due to its strong hydrophilicity and rapid water uptake. After stirring with a glass rod, the Unmodified-BC membrane was disintegrated and redispersed in deionized water. When the Modified-BC membrane was soaked in deionized water for 2 h, the membrane appeared to remain intact after stirring with a glass rod (Figure S4c in the Supporting Information). Furthermore, after continuous treatment in boiling water for 2 h and stirring with a glass rod, the membrane appeared to remain intact (Figure S4d in the Supporting Information). The water uptake of the Modified-BC membrane was markedly reduced due to the consumption of the cellulose -OH groups and chemical condensation leading to siloxane bridges (Si-O-Si) and to grafting onto the BC surface through Si-O-C bonds (shown in Figure 1).

**Figure 2:**
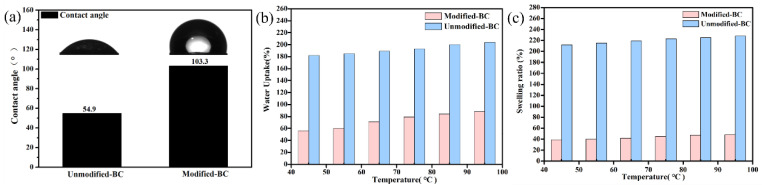
(**a**) Static water contact angles of Unmodified-BC and Modified-BC membranes; (**b**) water uptake of Unmodified-BC and Modified-BC membranes; (**c**) swelling ratios of Unmodified-BC and Modified-BC membranes.

The results above provide the possibility for a further application of the Modified-BC membrane to proton-exchange membrane fuel cells. In order to further study the membrane’s water stability, contact angle measurements were completed. As shown in Figure 2a, the contact angle of pristine bacterial cellulose was 54.9°, which is in line with the typical characteristics of pristine bacterial cellulose fiber. After modification with APTES, the contact angle of the Modified-BC membrane was more than 90° (103.3°, specifically), indicating hydrophobicity. Due to this hydrophobicity, it can still maintain a complete appearance after being immersed in deionized water for a period of time. As shown in Figure 2b, the water uptake of the Unmodified-BC membrane was close to 200%, resulting in its redispersion in water. However, the water uptake for the BC membrane modified with APTES was significantly reduced. Concretely, the water uptake of the Modified-BC membrane was 56% at 45 °C, about 30% that of the Unmodified-BC membrane. Similarly, the swelling of the Modified-BC membrane was significantly reduced from 212% to 39% at 45 °C (see Figure 2c). Herein, a law is proposed that the water uptake of the membrane increases with increasing temperature.

### 3.2. Appearance and Morphology

**Figure 3:**
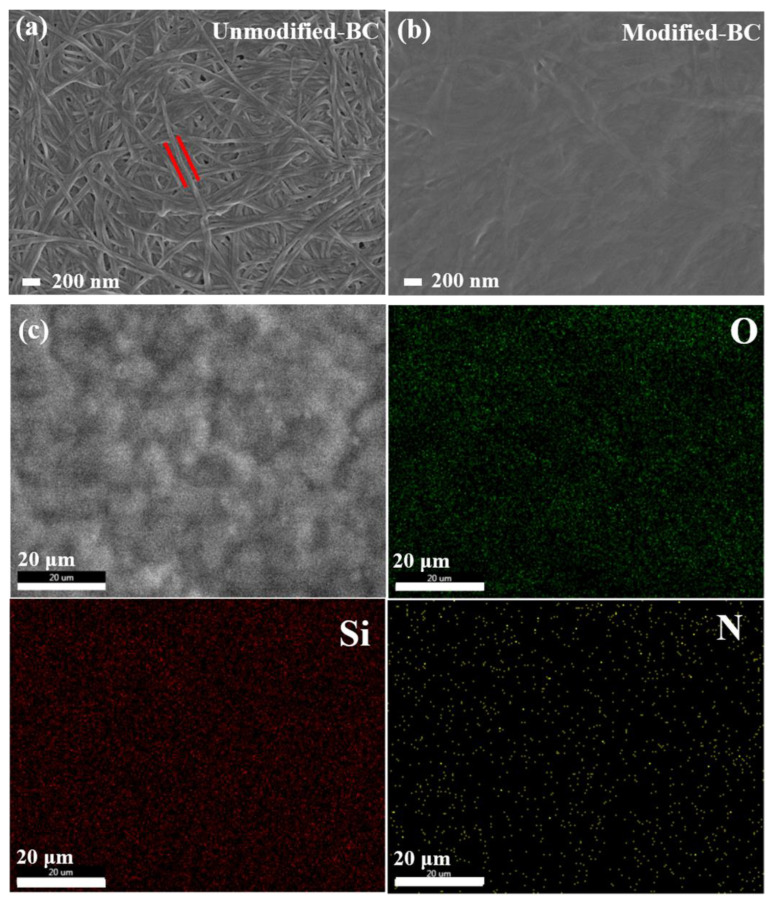
(**a**) SEM image of the surface of the Unmodified-BC membrane, ×20,000; (**b**) SEM image of the surface of the Modified-BC membrane, ×20,000; (**c**) SEM-EDS analysis of the Modified-BC membrane.

In this study, the chemical grafting of APTES onto BC involved three steps (shown in Figure 1): (i) the hydrolysis of the alkoxy groups of the amino silane in the presence of water to produce silanols; (ii) the adsorption of the silanol groups onto the -OH-rich surface of BC through hydrogen bonding between the silanol and the -OH groups; and (iii) chemical condensation leading to siloxane bridges (Si-O-Si) and to grafting onto the BC surface through Si-O-C bonds [29]. Figure 3a shows the surface morphology of the Unmodified-BC membrane, with a porous three-dimensional structure that is typical of bacterial cellulose fibers with diameters of 50~100 nm (shown as the distance between the two red strands) [30]. As shown in Figure 3b, the addition of APTES transformed these fibrils into a denser and more compact structure due to the deposition of the silane layer on the nanofiber backbone (Figure S5 in the Supporting Information). To confirm the elemental composition of the chemically modified membrane, SEM-EDS was utilized to obtain the distributions of silicon and nitrogen throughout the membrane, in addition to those of the main cellulose components (i.e., carbon and oxygen). As shown in Figure 3c, the main chemical elements (oxygen (O), silicon (Si), and nitrogen (N)) expected in the membrane were clearly detected, and the signal distribution was uniform. The distribution of carbon was omitted from Figure 3c. The main component elements of BC are carbon and oxygen, while APTES contains silicon and nitrogen. The presence of APTES in the Modified-BC Membrane was thus confirmed via the EDS analysis.

### 3.3. FT-IR Analysis

**Figure 4:**
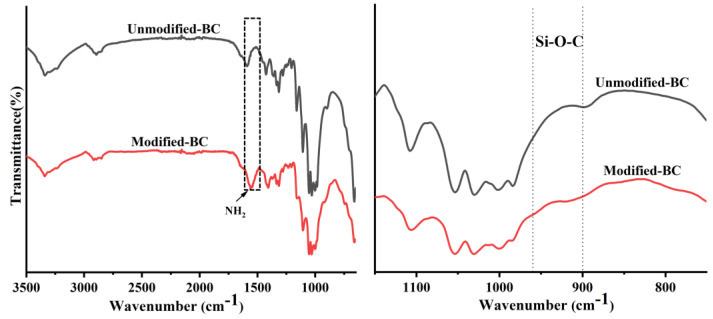
ATR−IR spectra of Modified−BC and Unmodified−BC membranes.

As shown in Figure 4, the main peaks associated with cellulose were observed for the Unmodified-BC and Modified-BC membranes, including O-H stretching at 3342 cm^−1^, C-H stretching at 2896 cm^−1^, and C-O stretching at 1032 cm^−1^ [31]. In addition, a characteristic peak at 1640 cm^−1^ was observed due to the presence of -OH groups from adsorbed water. After bacterial cellulose and water interact, it is difficult to completely remove the water adsorbed onto the cellulose molecules [32]. For the Modified-BC membrane, there were characteristic peaks unique to amino silane from APTES, in addition to the characteristic peaks of bacterial cellulose. There was also a new infrared peak at 1550 cm^−1^, which can be attributed to N-H bending vibrations. This peak indicated that amino groups were successfully grafted onto the surface of the bacterial cellulose [33]. Further evidence of the condensation reaction between bacterial nanocellulose and APTES was found in the spectral transition in the 900–960 cm^−1^ region, corresponding to the symmetric stretching and bending vibrations of Si-O-C [34].

### 3.4. XPS Analysis

**Figure 5:**
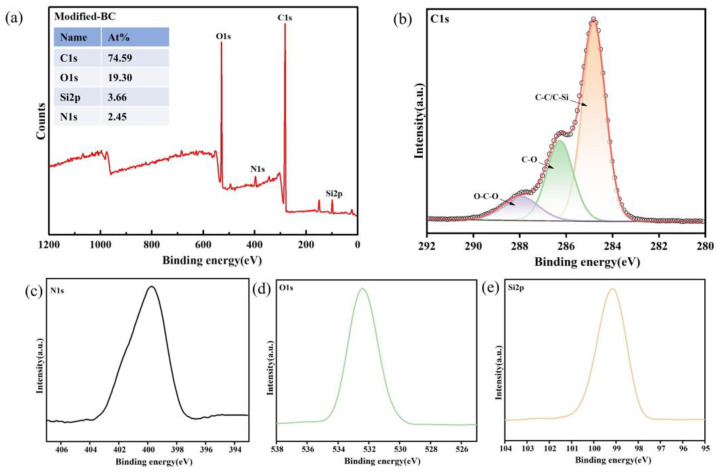
X-ray photoelectron spectroscopy of the Modified-BC membrane: (**a**) wide-scan spectrum; (**b**) C1s region; (**c**) N1s region; (**d**) O1s region; (**e**) Si2p region.

XPS was used to perform an elemental analysis. It is a suitable method to obtain qualitative and quantitative information about the different constituents of a surface and their chemical environment. The C1s, O1s, N1s, and Si2p photoelectron peaks for the Modified-BC membrane obtained via chemical modification with APTES are shown in Figure 5a. The results are consistent with the infrared spectrum and EDS measurement results (Figure 3 and Figure 4). As shown in Figure 5b, the C(1s) cladding of the Modified-BC membrane was composed of three components with a main peak at 286.2 eV (C-O bond), a higher-binding-energy O-C-O peak at 288 eV (acetal bond in bacterial cellulose), and a C-C or C-Si peak at 284.8 eV [35].

Following BC modification, the presence of nitrogen and silicon atoms was confirmed by the appearance of two new peaks in the survey spectrum at 399 and 99 eV, which were attributed to N1s and Si2p, respectively. This provides evidence of the successful introduction of APTES.

### 3.5. XRD Analysis

**Figure 6:**
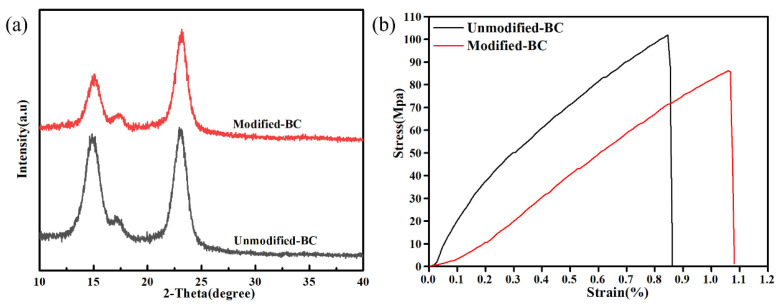
(**a**) X-ray diffraction spectra of Unmodified-BC and Modified-BC membranes; (**b**) stress–strain curves of Unmodified-BC and Modified-BC membranes (25 °C).

Chemical modifications to nanocellulose reduce the crystallinity of the initial fibers, thereby reducing their reinforcing effect [36]. Therefore, it is of great significance to retain the crystal structure of the initial nanocellulose fibers in the process of chemical modification. Cellulose is composed of a crystalline phase and, rarely, amorphous phases; the crystallinity of nanocellulose is defined as the mass fraction of the crystalline domain [37]. As shown in Figure 6a, no obvious difference was observed in the X-ray diffraction patterns for the Modified-BC and Unmodified-BC membranes. All the samples showed characteristic peaks at about 15°, 17°, and 23°, corresponding to the Miller index values of cellulose, i.a., (100), (010), and (110), respectively [38]. The results showed that the crystal structure of the Modified-BC membrane changed insignificantly. However, the intensity of the peaks for the Modified-BC membrane was decreased. As shown in Figure 6b, the tensile strength of the Modified-BC membrane was lower than that of the Unmodified-BC membrane due to the addition of APTES, which reduced the strength of the hydrogen bonding network between the nanofibers. However, the tensile strength (nearly 86 Mpa) of the Modified-BC membrane was still significantly higher than that of Nafion (30.7 ± 0.4 MPa) [39]. In general, the tensile strength of the Modified-BC membrane can still meet or even exceed the requirements of proton-exchange membranes for PEMFCs.

### 3.6. Chemical Stability

The chemical stability of the membrane samples was studied by using a Fenton experiment to simulate the fuel cell operating environment [17]. Concretely, samples were dried in a vacuum at 80 °C for 4 h and measured using an analytical balance (±0.1 mg). The samples were then immersed in Fenton solution (5 ppm Fe^2+^, 3 wt% H_2_O_2_) for 1 h at 80 °C. Subsequently, the samples were washed three times with deionized water and vacuum-dried at 80 °C for 4 h. The weight loss of the membrane samples after the Fenton treatment was measured. The initial BC membrane was completely dissolved due to its high hydrophilicity. The BC membrane modified with APTES maintained 96% of its mass during the test (Table S1 in the Supporting Information), indicating that the Modified-BC membrane had good chemical stability.

### 3.7. Proton Conductivity

**Figure 7:**
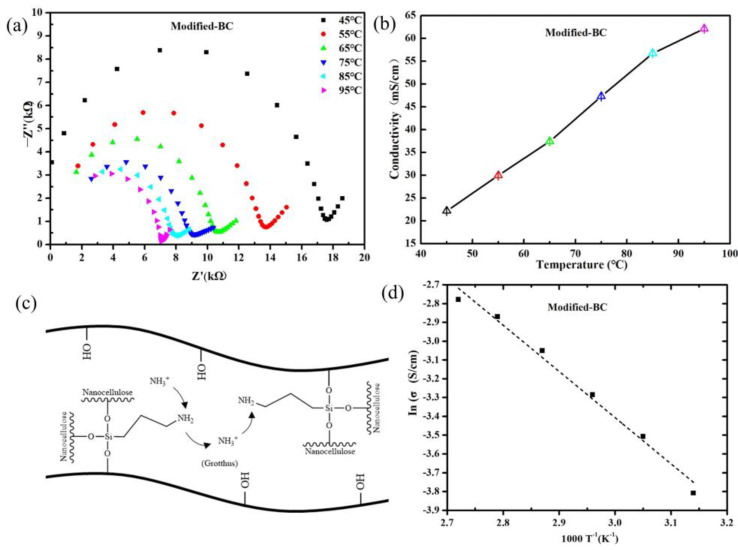
(**a**) Electrochemical impedance curves of Modified−BC membrane; (**b**) dependence of proton conductivity for Modified−BC membrane at 100% RH and different temperatures (45 °C to 95 °C); (**c**) Grotthuss−like mechanism of proton conductivity for Modified−BC membrane; (**d**) Arrhenius plot of Modified−BC membrane.

The proton-conducting behavior of the Modified-BC membrane was investigated by means of electrochemical impedance spectroscopy. As already reported in other studies [25], bacterial nanocellulose is a poor ionic conductor, with conductivity (σ) values ranging from 1.7 × 10^−7^ mS/cm at 40 °C and 40% RH to 6.3 × 10^−2^ mS/cm at 94 °C and 98% RH. As shown in Figure 7b, the proton conductivity increased with increasing temperature, which is consistent with the typical characteristics of PEMs [6]. The optimal ratio for the preparation of the membrane was identified as the one that achieved the maximum protonic conductivity, which was observed to be 62.2 mS/cm at 100% and 95 °C when APTES was combined with bacterial cellulose. The proton conductivity’s dependence on temperature was proved. Figure 7d demonstrates the linear Arrhenius-type behavior of this dependence: $\sigma =\sigma_{0}*e^{-E_{a}/RT}$, where $\sigma_{0}$ is a pre-exponential term,$\;E_{a}$ is the activation energy for ion transport, R is the gas constant, and T is the absolute temperature [40]. The activation energy for the Modified-BC membrane was 20.46 KJ/mol, which was determined from the slopes of the Arrhenius plot and can provide valuable information about which conduction mechanism is responsible for the membrane’s proton conduction. The proton conductivity (62.2 mS/cm) of the Modified-BC membrane was within the range consistent with the Grotthuss-like mechanism, viz., 10–50 kJ/mol, making this the most likely conduction mechanism [22,41]. In the Grotthuss mechanism, protons jump between adjacent sulfonic acid groups, hydroxyl groups, or water molecules [14,42]. Water molecules act as carriers for the formation of transition hydrogen bonds during this proton hopping. The protons produced by anodic fuel through an oxidation reaction combine with H_2_O to form transition hydronium ions by means of hydrogen bonding. The introduction of these additional protons results in the destruction of the transition hydronium ions, the disruption of hydrogen bonds, and the dissociation of protons, which are then transferred to the surrounding ion sites through the formation of new hydrogen bonds, thereby completing the proton jump transfer [43,44,45]. In the Modified-BC membrane, the proton carrier (-NH_2_) plays an important role in the transfer of protons. Firstly, the functional groups (-NH_2_) in the Modified-BC membrane form N-H⋯O and O-H⋯N hydrogen bonds with -OH groups (or possibly residual water) on the cellulose surface [46,47]. Subsequently, the protons generated via the oxidation of the anode fuel (H_2_) combine with the -NH_2_ groups as proton acceptors, forming -NH_3_^+^ cations. To maintain the charge balance, the vicinity of the positive -NH_3_⁺ ions is filled with OH^−^ ions, and NH_3_^+^⋯OH^−^⋯H^+^ transition intermediates form through hydrogen bonding. Thereafter, the proton dissociates and binds to the downstream proton carrier, completing the proton transfer.

### 3.8. Fuel Cell Performance

**Figure 8:**
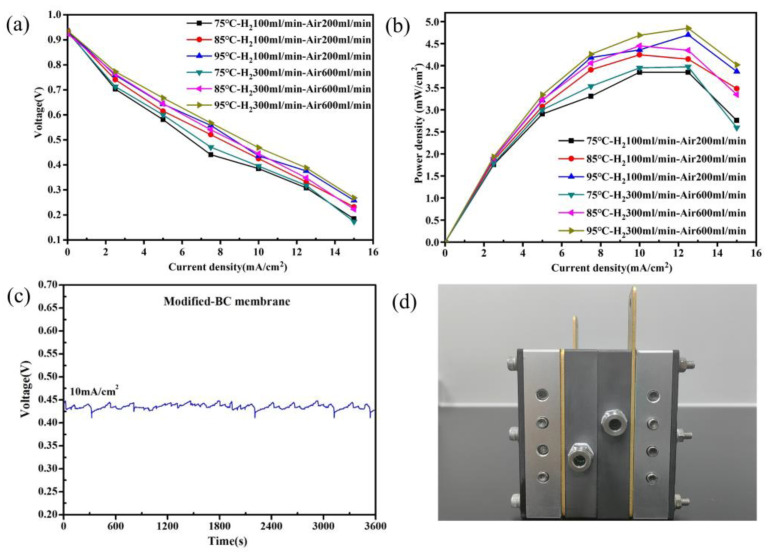
(**a**) IV curves of a single fuel cell at 100% RH; (**b**) power density curves of a single fuel cell at 100% RH; (**c**) stability of the output voltage in a single fuel cell at 100% RH and 85 °C; (**d**) photo of the fuel cell membrane electrode test fixture.

On the basis of the favorable results above, the Modified-BC membrane was used to fabricate an MEA. The catalyst layer on both sides of the membrane was sprayed using a 260E ultrasonic spraying system (Figure S6 in the Supporting Information). The catalyst loadings on the anode and cathode were 0.4 mg Pt/cm^2^ and 0.2 mg Pt/cm^2^, respectively. Then, two gas diffusion layers were composited with the membrane. Herein, a 4 cm^2^ MEA was prepared. The MEA was installed in a single-cell fixture (Figure 8a), and its performance was measured using fuel cell testing equipment. Subsequently, the polarization curves, power density plots, and stability were investigated. As shown in Figure 8b, the open-circuit voltage (OCV) of the Modified-BC membrane was almost 0.92 V. Although this is about 50 mV lower than that of conventional membranes [39], it shows that the nanocellulose membrane modified with APTES is insulated and meets the requirements for insulation and gas barriers in PEMFCs. The maximum power density reached 4.85 mW/cm^2^ when a current was loaded at 100% RH and 95 °C, with H_2_ and air at flow rates of 300 mL/min and 600 mL/min, respectively. Further, the stability of the output voltage for 1 h was evaluated by loading a current density of 10 mA/cm^2^ at 100% and 85 °C, with a H_2_ flow of 300 mL/min and an air flow of 600 mL/min (Figure 8c). The data showed that the voltage amplitude was small, and the voltage was stable during continuous testing under the given constant current.

### 3.9. Cost Analysis

Although the performance of the Modified-BC membrane is undoubtedly lower than that of Nafion, this deficiency could be compensated for by its lower cost. The current price of the Nafion 212 membrane is 6000–8000 CNY/m^2^, while the cost of the Modified-BC membrane used in this study is nearly 760 CNY/m^2^. Even on a small scale, the cost of the Modified-BC membrane is reduced by almost 10 times. Large-scale preparation would further reduce the material costs. Additionally, as a biodegradable polymer, the Modified-BC membrane has advantages over fluorinated and hydrocarbon PEMs for disposable and short-term applications, such as most portable primary and secondary batteries in use today.

## 4. Conclusions

In this study, we sought to improve the performance of nanocellulose for applications as ecofriendly and low-cost proton-exchange membranes in PEMFCs. To this end, APTES containing -NH_2_ functional groups was introduced to nanocellulose membranes to simultaneously improve their proton conductivity and water stability. The FTIR, SEM, EDS, and XPS data confirmed the success of the silanization reaction. The results revealed that the Modified-BC membrane prepared by using the optimal ratio of BC and APTES solved the problem of BC membrane dispersion in water; it also had a good proton conductivity of 62.2 mS/cm and generated a power performance of 4.85 mW/cm^2^ when used in a PEMFC. However, although the proton conductivity was high in the plane direction, the performance was not as high as expected in the tested PEMFC. This may be due to the lack of an obvious nano–microphase separation proton channel, which is not conducive to the vertical direction of proton conduction. To verify this supposition, the small-angle X-ray scattering (SAXS) patterns of the Modified-BC membrane were obtained. The results showed that there was no discernible nano–microphase separation in the APTES-modified BC membrane due to the absence of a clear ionomer peak (Figure S7 in the Supporting Information). This provides a possible research direction for the future.

Whilst the power density of the Modified-BC membrane was significantly lower than that of the conventional Nafion membrane, its lower cost and biodegradability provide advantages for some applications, such as biodegradable electronics and short-term fuel cells that are cheap enough to even be disposable, much like most portable primary and secondary batteries today. To the best of our knowledge, this work provides the first example where the simple addition of APTES with an alkaline proton carrier is proposed as a low-cost method to obtain a fully bio-based conductive membrane; we hope to inspire future research on and the applications of cellulose proton-exchange membranes in PEMFCs.

There are some remaining problems that are worthy of attention in future research, including the service period of the developed cellulose membranes, their use in various operating conditions, and more in-depth studies on their chemical stability. Briefly, although challenges still exist in terms of practical applications, the demonstrated ability to adjust the properties of cellulose via molecular engineering promises to drive the development of cellulose-based PEMFCs for electronics with high performance and stability.

## Data Availability

The original contributions presented in the study are included in the article/Supplementary Material, further inquiries can be directed to the corresponding author.

## Supplementary Materials

The following supporting information can be downloaded at: https://www.mdpi.com/article/10.3390/polym16213017/s1.
