# Supplementary material for: A Sustainable and Eco-Friendly Membrane for PEM Fuel Cells Using Bacterial Cellulose
Source: Polymers (Basel). 2024 Oct 28;16(21):3017. doi: 10.3390/polym16213017 (PMC11548236; doi:10.3390/polym16213017)
Supplement: Supplementary file 1 [file polymers-16-03017-s001.zip › polymers-3235085-supplementary.pdf]

A Sustainable and Eco-Friendly Membrane for PEM Fuel Cells using Bacterial Cellulose

Supporting Information

Xiaozhen Yang, Lin Huang, Qiang Deng, Weifu Dong*

The Key Laboratory of Synthetic and Biological Colloids, Ministry of Education, School of Chemical and Material Engineering, Jiangnan University, 1800 Lihu Road, Wuxi 214122, China

E-mail: wfdong@jiangnan.edu.cn

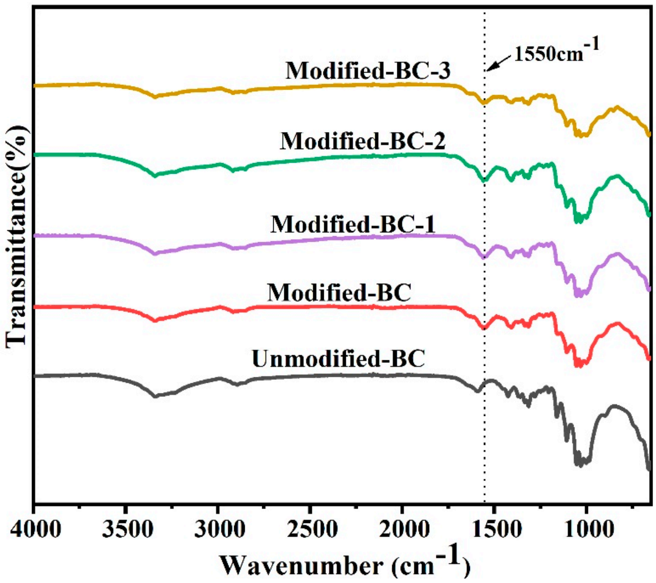

Figure S1. ATR-IR spectra of membrane samples.

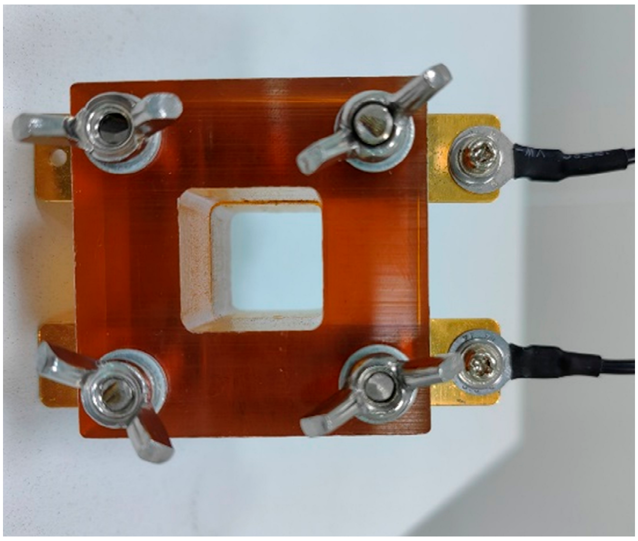

Figure S2. Photo of a test fixture for proton conductivity.

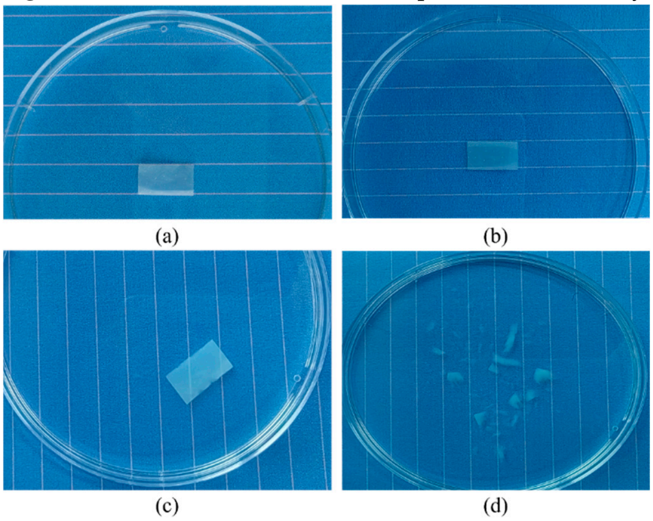

Figure S3. Photo of Unmodified-BC membrane. **(a)** Dry; **(b)** Immediately after immersion in deionized water; **(c)** 5 min after immersion in deionized water; **(d)** 5 min after immersion in deionized water by stirring with a glass rod.

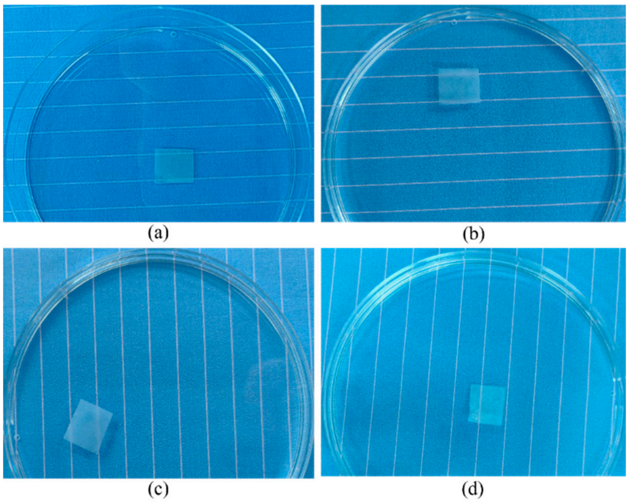

Figure S4. Photo of Modified BC membrane. **(a)** Dry; **(b)** Immediately after immersion in deionized water; **(c)** 2 h after immersion in deionized water by stirring with a glass rod; **(d)** 2h after continuing boiled with deionized water by stirring with a glass rod.

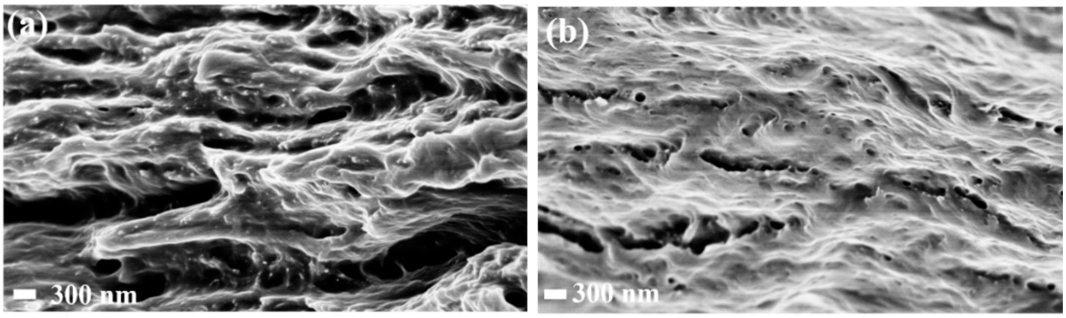

Figure S5. Scanning electron microscopy images for the cross-section. **(a)** Unmodified-BC membrane, ×15,000; **(b)** Modified-BC membrane, ×15,000.

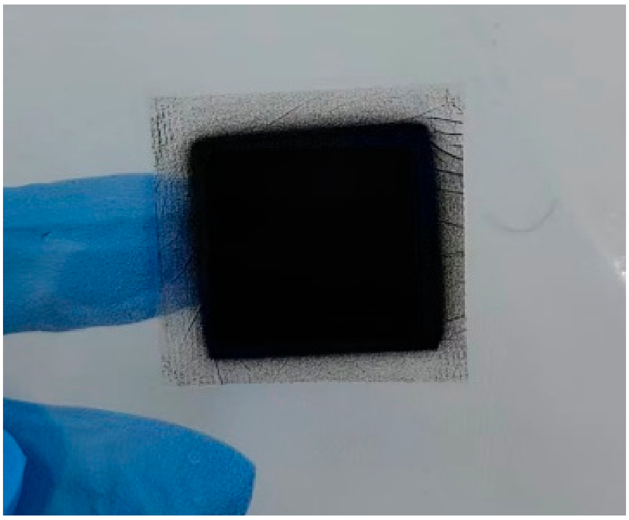

Figure S6. The catalyst layer was coated on the Modified-BC membrane.

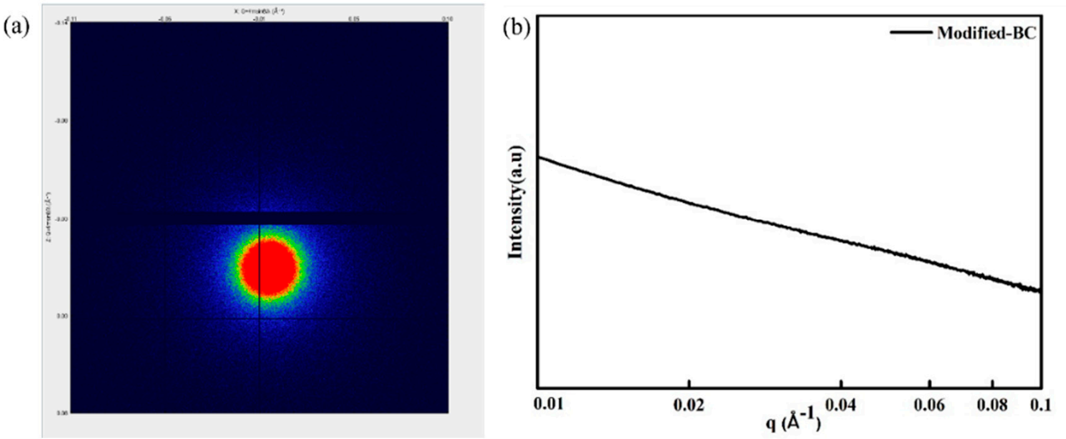

Figure S7. (a) The SAXS patterns of the Modified-BC membrane (sample to detector distance 1500 mm, etched for 10 min) recorded; **(b)** The intensity profiles of the scattering.

Table S1. The Fenton's test at 80℃ for Modified-BC membrane chemical stability

Sample	Initial mass (g)	Fenton's test time	Mass after test (g)	Weight loss
1	0.0396	1h	0.0380	4.0%
2	0.0378	1h	0.0364	3.7%
3	0.0388	1h	0.0371	4.4%

Table S2. Summary of various cellulose based membranes utilized so far in proton exchange membrane fuel cells (PEMFCs)

Cellulose	Process and modifications	Proton Conductivity	Ref.
Bacterial Cellulose	Crosslinked with fucoidan	1.7×10^{-10} S/cm (40℃, 40%) 6.3×10^{-5} S/cm (94℃, 98%).	[25]
	Polymerized in-situ with poly (4-styrene sulfonic acid) (PSSA) to form nanocomposite	5.4 mS/cm (25℃, 98%).	[26]
Nanocrystal Cellulose	Crosslinked with sulfosuccinic acid	15 mS/cm (fully hydrated state, 120∘C)	[27]
Cellulose Nanofibers	Carboxylation	0.0015 mS/cm (30℃, 95% RH)	[20]
	Sulfonation	2×10^{-3} S/cm (120℃, 100% RH)	[17]
	Crosslinked with citric acid	9.4 mS/cm 80℃	[28]
Cellulose Acetate	Graphene oxide	0.0155 S/cm (RT, 80%-85%RH)	[29]
